# Modeling and Analysis of Phase Fluctuation in a High-Precision Roll Angle Measurement Based on a Heterodyne Interferometer

**DOI:** 10.3390/s16081214

**Published:** 2016-08-02

**Authors:** Junhui Huang, Zhao Wang, Jianmin Gao, Bao Yu

**Affiliations:** 1 School of Mechanical Engineering, Xi’an Jiaotong University, Xi’an 710049, China; yubao.123@stu.xjtu.edu.cn; 2 State Key Laboratory for Manufacturing Systems Engineering, Xi’an Jiaotong University, Xi’an 710049, China; gjm@mail.xjtu.edu.cn

**Keywords:** roll angle, heterodyne interferometer, phase metering, phase fluctuation, modeling

## Abstract

Heterodyne interferometry is a high-precision method applied in roll angle measurements. Phase metering is essential for high precision. During a high-precision measurement, a phase fluctuation appears even when the roll angle does not vary, which has never been analyzed before. Herein, the reason for the phase fluctuation is revealed, which results from the frequency-difference fluctuation and time difference between measurement and reference beams. A mathematical model of that phase-fluctuation mechanism is established, and that model provides a theoretical basis for analyzing and reducing the phase fluctuation. The impact that the main factors have on the phase metering is analyzed quantitatively, and experiments are carried out to validate the model. Finally, the phase fluctuation decreases to 0.02° by frequency reduction, which conversely verifies the theoretical model.

## 1. Introduction

**Figure 1:**
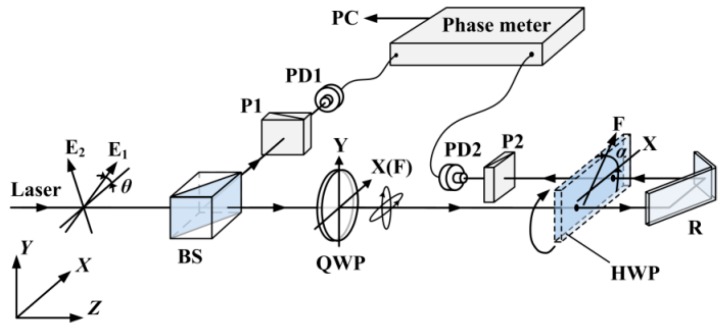
Schematic principle for a high-precision small roll angle measurement.

The heterodyne interferometer has been a widely applied metrological instrument for displacement measurements for the past 40 years [1,2,3,4,5,6]. It affords the advantages of high resolution, high noise immunity, and easy realization. Because it has recently become necessary to accurately measure the angular displacement measurement of a worktable in a high-end CN machine tool, the heterodyne interferometer has been also used to achieve a high-precision roll angle measurement [7,8,9,10,11,12]. A high-sensitivity measurement scheme for a small roll angle is shown in Figure 1, in which a phase retarder such as a half-wave plate (HWP) is adopted as the sensor for the roll angle test and a phase shift of the measurement signal and the reference signal is detected by a phase meter to demodulate the roll angle [8,9,10,11,12]. Theoretically, a subsecond precision angle measurement can be achieved by adopting a high-precision phase meter [8,9,11,12]. However, it is difficult to achieve such a high precision in practice because the precision of the phase metering is obstructed by various errors. In the past, various error sources causing phase errors have been investigated, such as the imperfection of polarizing beam splitters, misalignment of their optical setup, amplitude variation in coherent transmission, multiorder Doppler frequency shift, elliptical polarization, and nonorthogonality of light beams [13,14,15,16,17,18,19,20]. These error sources cause a periodic nonlinear error. However, in the roll angle measurement, the nonlinearity of the phase is utilized to increase the sensitivity, and it has little influence on precision because the relationship between the roll angle and the phase shift can be approximated as linear in such a small measurement range (1° or less) [8,11,12]. Even so, the precision of the phase metering is required to be higher for high-precision roll angle measurements, and in many practical experiments, we have found that the phase shift fluctuates continuously even when the roll angle does not vary. That small fluctuation may be easily ignored in the general phase metering, but it has a significant impact in high-precision phase metering. It cannot be directly interpreted by air turbulence or other environmental factors or the repeatability of the phase-detecting electronics, because its fluctuation period is the same as the frequency fluctuation, which is mainly resulted from frequency fluctuation. Frequency fluctuation is a common phenomenon and can reach tens or hundreds of kHz, which has been also observed by other scholars [21,22]. These authors presented a dynamic base signal with a phase-locked loop to track the changing frequency in their frequency mixing and frequency reduction, but they did not mention the phase fluctuation, nor did they investigate the reason or mechanism. In high-precision phase metering, small phase fluctuation cannot be ignored. This study reveals the main factors causing the phase fluctuation and models its mechanism; based on the model, the factors and their impacts on phase metering are analyzed quantitatively. 

The organization of the paper is as follows. In Section 2, the principle underlying roll angle measurements is described. It is the theoretical basis for modeling the phase fluctuation. In Section 3, the factors resulting in additional phase shifts are presented and a mathematical model of additional phase shifts is established; then, through theoretical derivation and simplification, the main factors are revealed and the mathematical model of the phase fluctuation is obtained. In Section 4, experiments are carried out to validate the mathematical model. Finally, conclusions are summarized in Section 5.

## 2. Roll Angle Measurement Based on a Heterodyne Interferometer

As shown in Figure 1, a beam from a double-frequency laser containing two orthogonal linear polarization components (E1 and E2) with a slight frequency difference applied to beat frequency is split into two beams by a nonpolarizing beam splitter (BS). The reflected beam is used as the reference signal after passing through a polarizer (P1) and being received by a photodetector (PD1). The transmitted beam passes through a quarter wave plate (QWP) and becomes slightly elliptically polarized by controlling the azimuth of the QWP. Moreover, it passes a half-wave plate (HWP), which is employed as the sensor for the roll angle measurement. Then it is rebounded by a reflector (R) and passes the HWP again. Ultimately, it is received by another photodetector (PD2) after passing through another polarizer (P2), and used as the measurement signal. The roll angle of the HWP can be demodulated by a phase shift between the two signals. 

The measurement signal ${\vec{E}}_{M}$ can be expressed based on the Jones matrix as:

(1) $${\vec{E}}_{M}=PH(-\alpha )H(\alpha )QR(\theta ){\vec{E}}_{0}$$

where ${\vec{E}}_{0}$ is the initial state and ${\vec{E}}_{0}=\left[\begin{array}{c} E_{1}\\ E_{2} \end{array}\right]=\left[\begin{array}{c} A_{1}e^{-i(\omega_{1}t+\varphi_{1})}\\ A_{2}e^{-i(\omega_{2}t+\varphi_{2})} \end{array}\right]$, *R*(*θ*) is the Jones matrix of the rotation and $R(\theta )=\left[\begin{array}{cc} \cos\theta & -\sin\theta \\ \sin\theta & \cos\theta \end{array}\right]$ (the fast axis of the QWP is set as the *x* axis of the Cartesian coordinate system for convenient expression), *Q* is the Jones matrix of the QWP and $Q=\left[\begin{array}{cc} 1 & 0\\ 0 & i \end{array}\right]$, *H*(*α*) is the Jones matrix of the HWP and $H(\alpha )=\left[\begin{array}{cc} \cos^{2}\alpha & \sin\alpha \cos\alpha \\ \sin\alpha \cos\alpha & \sin^{2}\alpha \end{array}\right]$, *P* is the Jones matrix of the polarizer and $P=\left[\begin{array}{cc} 1 & 0\\ 0 & 0 \end{array}\right]$, *α* is the angle between the fast axis of the HWP and *x* axis that is regarded as the roll angle under test, and *θ* is the angle between the electric vector E1 and the fast axis of the QWP.

Substituting the above matrices into Equation (1) and then simplifying gives:

(2) $${\vec{E}}_{M}=\left\{k_{1}A_{1}e^{-i[2\pi f_{1}t+\varphi_{1}-\arctan(\tan\theta \tan(4\alpha ))]}+k_{2}A_{2}e^{-i[2\pi f_{2}t+\varphi_{2}-\arctan(\cot\theta \tan(4\alpha ))]}\right\}\left[\begin{array}{c} 1\\ 0 \end{array}\right]$$

where $k_{1}=\sqrt{\cos^{2}\theta \cos^{2}(4\alpha )+\sin^{2}\theta \sin^{2}(4\alpha )}$ and $k_{2}=\sqrt{\sin^{2}\theta \cos^{2}(4\alpha )+\cos^{2}\theta \sin^{2}(4\alpha )}$.

According to Equation (2), the intensity *I_M_* is written as

(3) $$I_{M}=\left|{\vec{E}}_{M}\right|=k_{1}^{2}A_{1}^{2}+k_{2}^{2}A_{2}^{2}+2k_{1}k_{2}A_{1}A_{2}\cos[2\pi (f_{1}-f_{2})t+\varphi_{1}-\varphi_{2}+\psi ]$$

The last item *ψ* is the phase shift caused by the roll angle and

(4) ψ=−[arctan(tanθtan(4α))+arctan(cotθtan(4α))]

Similarly, ${\vec{E}}_{R}=PR(\theta ){\vec{E}}_{0}$ and the intensity *I_R_* of the reference signal can be written as:

(5) $$I_{R}={\left|{\vec{E}}_{R}\right|}^{2}=A_{1}^{2}+A_{2}^{2}+2A_{1}A_{2}\cos[2\pi (f_{1}-f_{2})t+\varphi_{1}^{'}-\varphi_{2}^{'}]$$

**Figure 2:**
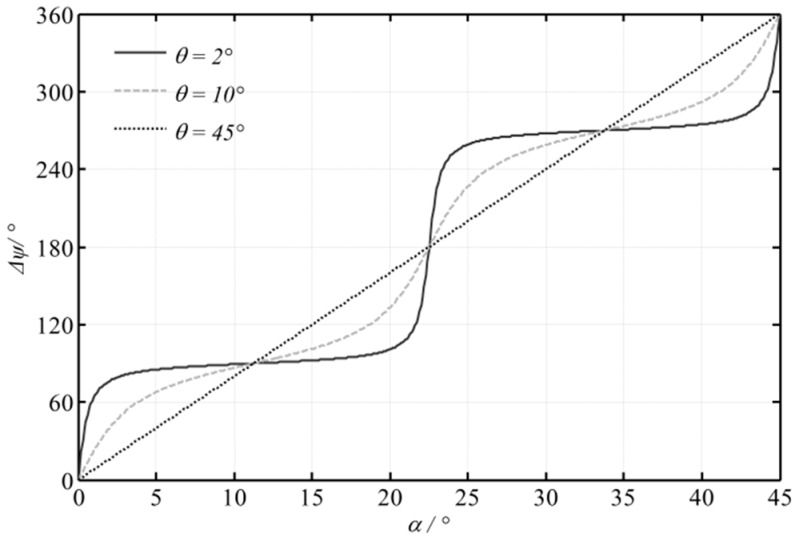
Theoretical ∆*Ψ*-*α* curves with differing *θ*.

*I_M_* and *I_R_* are beat signals whose frequencies are the frequency difference between E1 and E2. They are extracted to AC signals instead of DC signals for phase shift metering. Then the phase shift ∆*ψ* between measurement and reference signals is given as:

(6) $$\Delta \psi =\varphi_{0}-[\arctan(\tan\theta \tan(4\alpha ))+\arctan(\cot\theta \tan(4\alpha ))]$$

where $\varphi_{0}=(\varphi_{1}-\varphi_{2})-(\varphi_{1}^{'}-\varphi_{2}^{'})$ is a constant during the measurement. Generally, the relationship between ∆*ψ* and *α* is nonlinear unless θ = 45°. Figure 2 shows the relationship between ∆*ψ* and *α* with differing θ. However, in a small range, a nearly linear segment can be found and it has a steep slope, which provides high sensitivity. Then, the relationship of ∆*ψ* and *α* is simplified as [8,11,12]:

(7) $$\Delta \psi =K_{\alpha }\alpha$$

where *K_a_* is the slope of the local segment defined as the system magnification, and $K_{\alpha }\approx -4(\tan\theta +\cot\theta )$. 

According to Equation (7), the roll angle *α* can be obtained by metering the phase shift ∆*ψ*, and the precision mainly depends on the precision of phase metering. When the azimuth of QWP *θ* is set to about 2°, the system magnification will reach *K_a_* ≈ 114.

## 3. Modeling and Analysis of the Phase Fluctuation

Theoretically, the phase items containing the frequency difference (*f*_1_ − *f*_2_) in Equations (3) and (5) can be subtracted. However, an additional phase shift would be generated because of the frequency fluctuation and the time difference of the signal response between the measurement and reference beams. Here, it is discussed below in detail.

### 3.1. Model of Additional Phase Shifts

**Figure 3:**
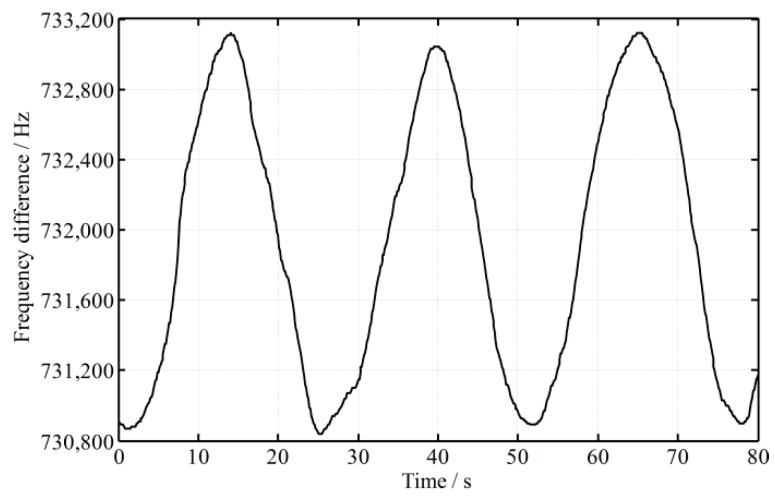
Fluctuation of the frequency difference of a double frequency laser.

In practice, temperature, atmospheric change, mechanical vibration, magnetic field, etc., all of these will cause the frequency instability, which result in the frequency difference (*f*_1_ − *f*_2_) constantly changing. The fluctuation of the frequency difference of a He-Ne Zeeman dual frequency laser is shown in Figure 3. From Figure 3, it is known that the frequency difference changes periodically and approximately sinusoidally over time, so the frequency difference can be described as:

(8) $$f_{1}-f_{2}=f_{0}+A_{0}\sin(2\pi f_{c}t+\varphi_{c})$$

where *f*_0_ is an offset, *A*_0_ is the amplitude of the fluctuation, *f_c_* is the frequency of the frequency-difference fluctuation, and *ϕ_c_* is the initial phase. Even the fluctuation of (*f*_1_ − *f*_2_) is not the same as shown in Figure 3. The function of (*f*_1_ − *f*_2_) can also be written as a summation of a series of sinusoidal functions through a Fourier series except with a random variation, that is:

(9) $$f_{1}-f_{2}=F(f_{1}-f_{2})=f_{0}+\sum_{i=1}^{\infty }A_{0i}\sin(i\cdot 2\pi f_{c}t+\varphi_{ci})$$

Then, the main elements with large amplitude are selected to analyze their effects, and as a consequence, the sinusoidal function is a universal model expressing the frequency-difference fluctuation.

Usually, the frequency difference (*f*_1_ − *f*_2_) in the measurement signal and the reference signal can be considered to be synchronous, but practically the measured and reference frequencies differ because a different time delay between two beams that makes the (*f*_1_ − *f*_2_) asynchronous. For a common photodetector, it mainly contains photoelectric conversion, a bias circuit, a current/voltage converter, amplifications, a filter, and so on. It would cost several microseconds for those procedures, and the time difference is estimated to reach as much as hundreds of nanoseconds between the two beams. Moreover, the length difference of the light path between the measurement beam and the reference beam would be up to 10 m or more, and the time delay between them will reach 34 ns. As a result, the arrival time at the two photodetectors is also different. This results in a frequency shift between the two beams after passing through the different paths and responses from different photodetectors. That is,

(10) $$\left\{ \begin{array}{l} {\tilde{I}}_{M}=2k_{1}k_{2}A_{1}A_{2}\cos[2\pi (f_{1}-f_{2})(t-t_{1})+\varphi_{1}-\varphi_{2}+\psi ]\\ {\tilde{I}}_{R}=2A_{1}A_{2}\cos[2\pi (f_{1}-f_{2})(t-t_{2})+\varphi_{1}^{'}-\varphi_{2}^{'}]\\ {\left(f_{1}-f_{2}\right)}_{M}=f_{0}+A_{0}\sin[2\pi f_{c}(t-t_{1})+\varphi_{c}]\\ {\left(f_{1}-f_{2}\right)}_{R}=f_{0}+A_{0}\sin[2\pi f_{c}(t-t_{2})+\varphi_{c}] \end{array}\right.$$

where *t*_1_ and *t*_2_ are the time delay after traversing the paths and responses by PD1 and PD2, respectively. From above, the phase shift between the measured signal and the reference signal is simplified as:

(11) $$\begin{array}{ll} \Delta \psi = & 2\pi f_{0}(t_{2}-t_{1})+2\pi A_{0}\{\sin[2\pi f_{c}(t-t_{1})+\varphi_{c}]-\sin[2\pi f_{c}(t-t_{2})+\varphi_{c}]\}t\\ & +2\pi A_{0}\{t_{2}\sin[2\pi f_{c}(t-t_{2})+\varphi_{c}]-t_{1}\sin[2\pi f_{c}(t-t_{1})+\varphi_{c}]\}+\varphi_{0}+\psi \end{array}$$

Set $\Delta t=t_{2}-t_{1}$, $\Delta f=A_{0}\{\sin[2\pi f_{c}(t-t_{1})+\varphi_{c}]-\sin[2\pi f_{c}(t-t_{2})+\varphi_{c}]\}$, and $\psi_{F}=2\pi A_{0}\{t_{2}\sin[2\pi f_{c}(t-t_{2})+\varphi_{c}]-t_{1}\sin[2\pi f_{c}(t-t_{1})+\varphi_{c}]\}$. ∆*t* is a time difference between two beams. ∆*f* is a frequency shift between two beams, namely, $\Delta f={\left(f_{1}-f_{2}\right)}_{M}-{\left(f_{1}-f_{2}\right)}_{R}$. *ψ_F_* is named as a direct fluctuation phase shift. Then, the phase shift ∆*ψ* is simplified as:

(12) $$\Delta \psi =2\pi f_{0}\Delta t+2\pi \Delta ft+\psi_{F}+\varphi_{0}+\psi$$

### 3.2. Model and Analysis of Phase Fluctuation

From Equations (11) and (12), it is known that any additional phase will not exist when ∆*t* = 0, but three additional phase items will be generated when ∆*t* ≠ 0. The first additional item (2*πf*_0_∆*t*) can be regarded as a constant when the system is stable; thus it does not cause fluctuations and usually is regarded as a part of the definite phase difference *ϕ*_0_. The second and third additional items, (2π∆*ft*) and *ψ_F_*, vary over time and generate fluctuation errors in the phase shift. 

For the second item, the frequency shift ∆*f* varies constantly, so the effect of a photodetector’s response on the phase shift over a period of time *t* is rewritten as:

(13) $$\begin{array}{ll} \psi_{\Delta f} & =2\pi \int_{0}^{t}\Delta fdt=2\pi \int_{0}^{t}A_{0}\{\sin[2\pi f_{c}(t-t_{1})+\varphi_{c}]-\sin[2\pi f_{c}(t-t_{2})+\varphi_{c}]\}dt\\ & =2\pi A_{0}\int_{0}^{t}2\cos[2\pi f_{c}t+\varphi_{c}-\pi f_{c}(t_{1}+t_{2})]\sin(\pi f_{c}\Delta t)dt \end{array}$$

Generally, the fluctuation of the frequency difference (*f*_1_ − *f*_2_) changes slowly after the laser becomes stable, for example, *f_c_* is only 1/25 Hz as shown in Figure 3. Accordingly, *πf*_c_∆*t* is too small, nearing 0, and $\sin(\pi f_{c}\Delta t)\approx \pi f_{c}\Delta t$. Then, Equation (13) is simplified as:

(14) $$\begin{array}{ll} \psi_{\Delta f} & \approx 2\pi A_{0}\Delta t\int_{0}^{t}\cos[2\pi f_{c}t+\varphi_{c}-\pi f_{c}(t_{1}+t_{2})]\text{d2}\pi f_{c}t\\ & =2\pi A_{0}\Delta t\sin[2\pi f_{c}t+\varphi_{c}-\pi f_{c}(t_{1}+t_{2})]-C \end{array}$$

where $C=2\pi A_{0}\Delta t\sin[\varphi_{c}-\pi f_{c}(t_{1}+t_{2})]$ is also a constant and can be regarded as a part of the *ϕ*_0_. Consequently, Equation (14) can be simplified as:

(15) $$\psi_{\Delta f}=2\pi A_{0}\Delta t\sin[2\pi f_{c}t+\varphi_{c}-\pi f_{c}(t_{1}+t_{2})]$$

The third item is rewritten as:

(16) $$\begin{array}{ll} \psi_{F} & =2\pi A_{0}\{t_{2}\sin[2\pi f_{c}(t-t_{2})+\varphi_{c}]-t_{1}\sin[2\pi f_{c}(t-t_{1})+\varphi_{c}]\}\\ & \approx 2\pi A_{0}\sqrt{t_{1}^{2}+t_{2}^{2}-2t_{1}t_{2}}\sin(2\pi f_{c}t+\varphi_{c}-\beta )\\ & =2\pi A_{0}\Delta t\sin(2\pi f_{c}t+\varphi_{c}-\beta ) \end{array}$$

where, $\tan\beta =\frac{t_{1}\sin(2\pi f_{c}t_{1})-t_{2}\sin(2\pi f_{c}t_{2})}{t_{1}\cos(2\pi f_{c}t_{1})-t_{2}\cos(2\pi f_{c}t_{2})}$ is also small and nearing 0. Combining Equations (15) and (16), the fluctuation phase shift ∆*ψ_F_* is:

(17) $$\begin{array}{ll} \Delta \psi_{F} & =2\pi A_{0}\Delta t\sin[2\pi f_{c}t+\varphi_{c}-\pi f_{c}(t_{1}+t_{2})]+2\pi A_{0}\Delta t\sin(2\pi f_{c}t+\varphi_{c}-\beta )\\ & =2\pi A_{0}\Delta t\cdot 2\sin[2\pi f_{c}t+\varphi_{c}-\frac{\beta +\pi f_{c}(t_{1}+t_{2})}{2}]\cos[\frac{\beta -\pi f_{c}(t_{1}+t_{2})}{2}]\\ & \approx 4\pi A_{0}\Delta t\sin(2\pi f_{c}t+\varphi_{c}) \end{array}$$

Furthermore, the initial phase *ϕ_c_* does not affect the amplitude of the phase fluctuation. For that reason, the fluctuation item of the phase shift ∆*ψ_F_* can be simplified as:

(18) $$\Delta \psi_{F}=\psi_{\Delta f}+\psi_{F}\approx 4\pi A_{0}\Delta t\sin(2\pi f_{c}t)$$

The above Equation (18) means that the amplitude of phase fluctuation is mainly caused by the amplitude of the frequency-difference fluctuation *A*_0_ and the time difference Δ*t* between the two beams, and the phase fluctuation is inevitable in high-precision measurement since even a slight time difference and frequency fluctuation would still result in an obvious phase fluctuation.

## 4. Experiments and Discussion

**Figure 4:**
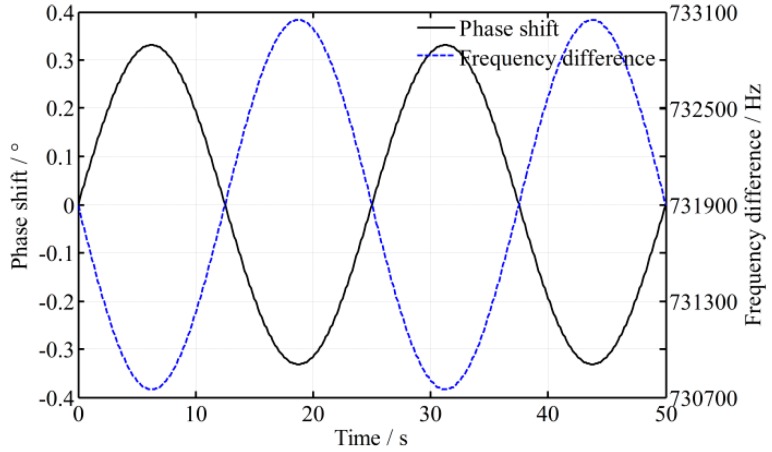
Simulation result of the phase shift and frequency difference varying over time.

**Figure 5:**
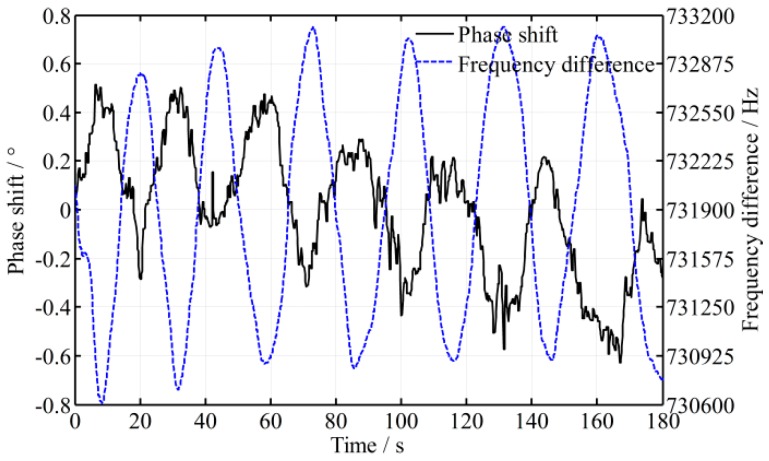
Practical result of the phase shift and frequency difference varying over time with two different model photodetectors.

To validate the mathematical model, an experimental platform is set up, as shown in Figure 1. Generally speaking, the time delay caused by difference in the lengths of the paths is much smaller than that caused by the responses of the photodetectors; in that case, only the time difference of photodetectors is considered in the following. As shown in Figure 3, the fluctuation period of the frequency difference 1/*f_c_* is about 25 s and its amplitude *A*_0_ is about 1.15 kHz. The intensity of the two beams is controlled to be slightly different, because when the intensity of measurement beam is smaller than 0.36 of the reference beam, the phase shift fluctuates dramatically. To illustrate the effect of time difference between the two beams, two photodetectors with different models are employed in the experimental platform, and the time difference of the signal processing ∆*t* is enlarged to be about 400 ns through simulation estimation. A high-accuracy phase meter with a resolution of 0.001° (Clarke-Hess CH6000A) is used for phase metering, and the length difference of the paths between the measurement and reference beams is controlled to be not more than 2 m. The fluctuation of frequency difference (*f*_1_ − *f*_2_) and its effect on phase ∆*ψ* is calculated through a Matlab simulation, and the simulation result as well as practical experiment data is shown in Figure 4 and Figure 5, respectively, where the roll angle is unchanged. It is noted that the orientation of the polarizer axis determines the sign of phase shift (positive or negative). The orientations of the polarizers would be different when two different model photodetectors are adopted and that results in the half cycle difference, as shown in Figure 5.

From Figure 5, it shows that the phase fluctuation varies sinusoidally and has the same period with a frequency difference (*f*_1_ − *f*_2_), which is entirely consistent with the theoretical model. Moreover, even a small time difference (400 ns) results in a significant phase fluctuation (0.70°). However, comparing with the practical curve of the phase fluctuation, it shows that the practical value is greater than the theoretical value, that is, the mean peak-to-peak value of the phase fluctuation with the practical data is about 0.70° more than the theoretical value 0.66°. That is because the time difference is not estimated accurately, and not only does the fluctuation of frequency difference disturb the phase metering, the environmental turbulence disturbs it too.

**Figure 6:**
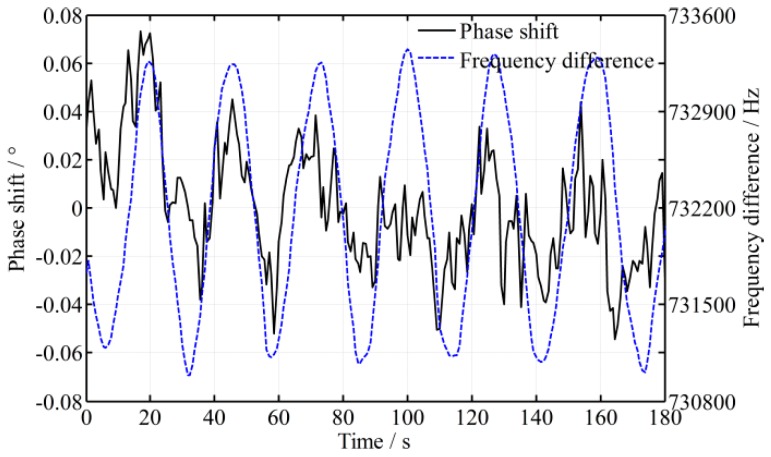
Phase shift and frequency difference varying over time with two same model photodetectors.

When *K_a_* = 114 and ∆*ψ_F_* = 0.70°, a fluctuation error of the roll angle test 22″ will be generated. It does not meet the high-precision of the roll angle measurement. Practically, two photodetectors of the same model are generally adopted in practical measurements. In this study, two photodetectors of the same model (Thorlabs PDA36A) are employed, and the experiment result is shown in Figure 6. Comparing with the results from two different model photodetectors as shown in Figure 5, the fluctuation of the phase shift is reduced to 0.10°, which is much less than the aforementioned 0.70°. That is consistent with the theoretical analysis, and the time difference is estimated to be reduced to about 100 ns. Obviously, the time difference affects the phase fluctuation greatly, even a slight time difference also results in an obvious phase fluctuation; and choosing the photodetectors with good consistency is the basis for high-precision phase metering.

**Figure 7:**
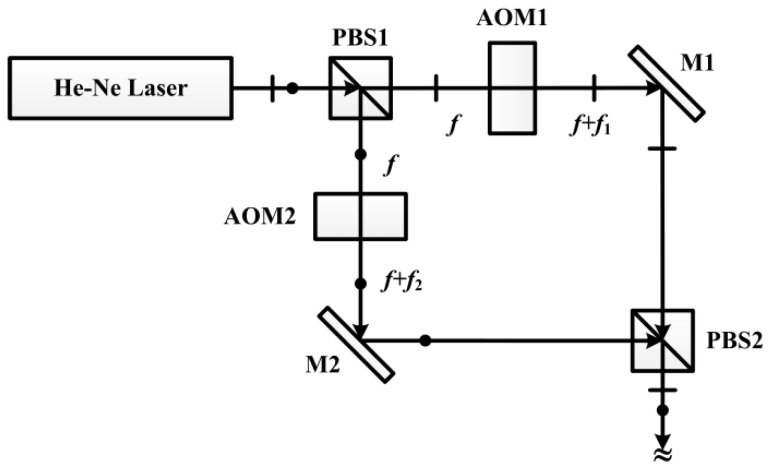
Generating a dual frequency orthogonal linearly polarized laser by two acousto-optic modulators.

In high-precision measurement, the above phase fluctuation is still too large, even if two same model photodetectors are employed. From the mathematical model, reduction of the fluctuation amplitude of the frequency difference is a way to reduce the phase fluctuation, but the frequency stability of one of the better commercial dual frequency lasers can only reach 1 kHz, which is still too large and would cause an obvious error in phase metering. Frequency reduction is verified to be an effective method to reduce the frequency fluctuation [21,22]. In this study, a stabilized He-Ne laser with two acousto-optic modulators (AOM) is adopted to generate dual frequency beams [9,23], wherein two AOM (Gooch and Housego AOMO 3080-125) with adjustable RF drivers (AODS Synth DDS 8 CH) are employed, as shown in Figure 7. Their center frequencies are 80 MHz, and the modulated frequencies are set to *f*_1_ = 80 MHz and *f*_2_ = 80.05 MHz, respectively, and a frequency difference of 50 kHz is obtained.

**Figure 8:**
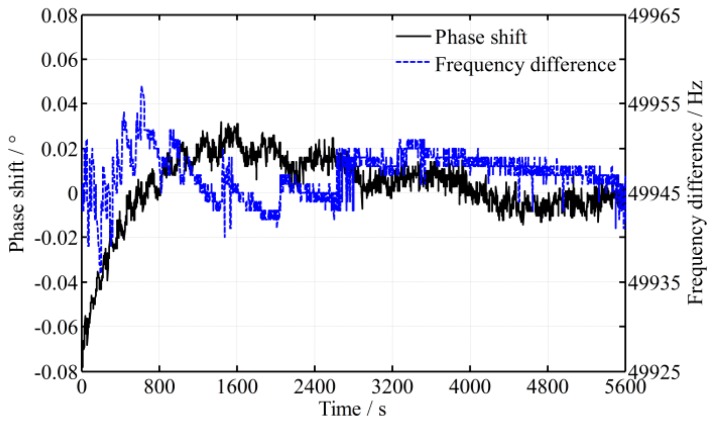
Phase shift and frequency difference varying over time with the acousto-optic modulator (AOM).

**Figure 9:**
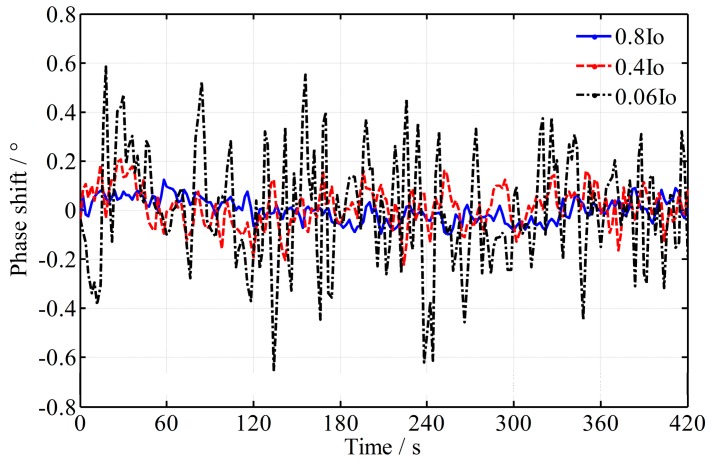
The influence of low light intensity on the phase fluctuation.

Detected by the high-precision phase meter, the variance of the phase shift and frequency difference is shown in Figure 8. It shows that the fluctuation of the frequency difference is reduced to less than 40 Hz and the fluctuation of the phase shift is reduced to about 0.02° when the laser is stable. This means that the fluctuation error of the roll angle test is reduced to 0.6’’ (*K_a_* = 114). For comparison, a simulation is performed and the theoretical peak-to-peak value of phase fluctuation is about 0.006°. The practical fluctuation is also much greater. That is because the beam intensity is reduced in the diffraction of the AOM, and it results in a decrease of photocurrent, which is more susceptible to noise and decreases the measurement accuracy [24]. Accordingly, the phase shift varies more randomly and the practical time difference ∆*t* would be larger than the aforementioned estimated value of 100 ns. Moreover, the influence of environmental turbulence can no longer be ignored. To verify the effect of light intensity on the phase fluctuation, an experiment is carried out, in which an optical attenuation slice is added in the measurement beam (in front of the photodetector) and the light intensity is reduced to 0.8*I*_0_, 0.4*I*_0_ and 0.06*I*_0_ respectively, where, *I*_0_ is the initial intensity of the measurement signal. The result is shown in Figure 9, where an additional fixed phase shift introduced by the optical attenuation slice is not considered and removed. From Figure 9, it shows that lower light intensity enlarges the phase fluctuation. Nevertheless, frequency reduction is still a valid way to reduce the phase fluctuation, which conversely verifies the theoretical analysis.

## 5. Conclusions 

This study reveals the reason of phase fluctuation and models the mechanism of the phase fluctuation caused by the fluctuation of the frequency-difference and the different time-delay between the measurement and reference beams in a heterodyne interferometer. Coming from the mathematical model, a fluctuation phase shift can be estimated quantitatively ahead of the measurement, and it shows that even a slight time difference could result in an obvious phase fluctuation. Experiments validate the mathematical model, and the experiment results indicate that frequency reduction is a valid way to reduce the phase fluctuation, which conversely verified the theoretical analysis. Ultimately, the phase fluctuation is reduced to 0.02°. Therefore, that mathematical model provides the theoretical basis for analyzing and reducing that phase fluctuation; it is especially important in a sub-second angle measurement. Furthermore, although the model is established based on the measurement scheme for a small roll angle, in fact, it is also suitable for the analysis of phase error, especially in picometer displacement measurements with a heterodyne interferometer.

## Abbreviations

The following abbreviations are used in this manuscript:

HWP	Half Wave Plate
QWP	Quarter Wave Plate
R	Reflector
P	Polarizer
PD	PhotoDetector
AOM	Acousto-Optic Modulator
